# Associations between preoperative american society of anesthesiologists physical status classification and postoperative outcomes in patients undergoing vertebroplasty

**DOI:** 10.1016/j.clinsp.2026.101145

**Published:** 2026-09-09

**Authors:** Chin-Chiu Chen, Heng-Hao Liu, Kun-Hui Chen, Ching-Heng Lin, Cheng-Hung Lee, Jun-Sing Wang

**Affiliations:** a Department of Anesthesiology, Taichung Veterans General Hospital, Taichung, Taiwan; b Department of Post-Baccalaureate Medicine, College of Medicine, National Chung Hsing University, Taichung, Taiwan; c Department of Orthopedics, Taichung Veterans General Hospital, Taichung, Taiwan; d Department of Computer Science and Information Engineering, Providence University, Taichung, Taiwan; e Department of Medical Research, Taichung Veterans General Hospital, Taichung, Taiwan; f Department of Food Science and Technology, Hung Kuang University, Taichung, Taiwan; g Division of Endocrinology and Metabolism, Department of Internal Medicine, Taichung Veterans General Hospital, Taichung, Taiwan; h Department of Music, Tunghai University, Taichung, Taiwan; i School of Medicine, College of Medicine, National Yang Ming Chiao Tung University, Taipei, Taiwan

**Keywords:** ASA classification, EuroQol-5D, Mortality, Oswestry disability index, Vertebroplasty

## Abstract

**Objective:**

The American Society of Anesthesiologists (ASA) physical status classification system is a preoperative evaluation regarding the risk of anesthesia during surgery. We investigated associations of ASA scores with postoperative Patient-Reported Outcome Measures (PROMs) and long-term survival rate in patients undergoing vertebroplasty.

**Methods:**

We retrospectively identified patients undergoing vertebroplasty for vertebral compression fractures from 2018 to 2020. PROMs were assessed using the Oswestry Disability Index (ODI) and EuroQol-5D (EQ-5D) prior to surgery and at postoperative follow-up. Patients were categorized by preoperative ASA scores (≤ 2 vs. ≥ 3). Between-group differences in postoperative changes of PROMs were compared. All-cause mortality was confirmed through March 2022. The association of ASA classification with all-cause mortality was examined using Cox-proportional hazard models.

**Results:**

A total of 199 patients were analyzed (ASA ≤ 2, n = 95; ASA ≥ 3, n = 104). Patients with an ASA ≤ 2 experienced a greater improvement in ODI (mean [SD] -35.8 [18.7] vs. -25.5 [17.4], p = 0.001) and EQ-5D (mean [SD] 0.42 [0.22] vs. 0.33 [0.26], p = 0.042) at 12-month follow-up than those having an ASA ≥ 3. After a median follow-up of 2.2-years, patients having a higher ASA score (≥ 3 vs. ≤ 2) experienced a greater risk of all-cause mortality (HR = 3.315, 95% CI 1.330 to 8.258, p = 0.010).

**Conclusion:**

A lower ASA score (≤ 2 vs. ≥ 3) was associated with a greater improvement in PROMs and better long-term survival after vertebroplasty for compression fractures. ASA classification may help identify high-risk patients, even in less invasive surgeries.

## Highlights

• Study subjects were patients undergoing vertebroplasty for compression fractures.
• Preoperative American Society of Anesthesiologists (ASA) score was assessed.
• Changes in postoperative Patient-Reported Outcome Measures (PROMs) were recorded.
• A lower ASA score (≤2 vs. ≥3) was associated with a greater improvement in PROMs.
• A lower ASA score (≤2 vs. ≥3) was associated with better long-term survival.

## Introduction

According to the World Health Organization (WHO), the population of people over 60-years old is estimated to reach 2.1 billion in the year 2050, which is double that of the year 2020. In Taiwan, the accelerated rate of aging is rising even faster, approximately twice that of European countries and the United States.^1^ The most common chronic diseases diagnosed among the elderly include hypertension and diabetes, with osteoporosis being a major issue for women. Since osteoporosis is often a “silent” disease before the occurrence of a compression fracture, the most common complications resulting from osteoporosis are often underrecognized.^2^ Osteoporosis may lead to compression fractures which sometimes require surgical intervention, including the minimally invasive spinal procedures vertebroplasty and kyphoplasty.^3^

Patient-Reported Outcome Measures (PROMs), such as the Oswestry Disbility Index (ODI)^4^ and EuroQol-5D (EQ-5D),^5^ are usually used after spinal surgery in order to assess patients’ outcomes.^6^ A prospective study^7^ revealed that vertebroplasty helped reduce self-reported pain scale results while also increasing activities of daily living. Additionally, similar outcomes were revealed in another comparative study^8^ showing improvement in both Visual analogue scale pain scores and the ODI post vertebroplasty or kyphoplasty. Controversially, a meta-analysis disclosed that though pain relief was found in the early period after vertebroplasty treatment, no statistical improvement in quality of life (EQ-5D being one of the assessment tools) was revealed.^9^ There still remains a lack of studies which show the relationship between PROMs and long-term prognosis in spinal surgery.

The American Society of Anesthesiologists (ASA) physical status classification system is a preoperative evaluation regarding the risk of anesthesia during surgery.^10^ The predictive value of ASA class and its association with postoperative outcomes in different surgeries has been studied with various results.^11^ Studies have shown ASA class to be significantly associated with post-operative complications,^12^ as well as morbidity and mortality,^13^^,^^14^ in spinal surgery patients. However, studies regarding the correlation between ASA class and both postoperative PROMs and long-term outcomes in patients undergoing vertebroplasty remain scarce. We aimed to investigate associations of ASA scores with postoperative PROMs and long-term survival rate in patients undergoing vertebroplasty.

## Materials and methods

We retrospectively identified patients who had been presented to our Department of Orthopedics with a single-level vertebral compression fracture between the years 2018 and 2020. These patients were admitted for a vertebroplasty after having undergone a pre-operative assessment. We excluded patients who had pathologic fractures, as well as those who had missing ASA physical status classification records. This study was conducted following the principles outlined in the Declaration of Helsinki. The study protocol was approved by the Institutional Review Board of Taichung Veterans General Hospital, Taichung, Taiwan (Approval number: CE24336B). The requirement for informed consent was waived due to the retrospective study design. This retrospective observational study was reported in accordance with the STROBE Statement.

Patients’ demographic variables including age, gender, body mass index, smoking history, and medical conditions, such as diabetes, hypertension, and chronic kidney disease, were all obtained from the available electronic medical records. ASA classification scores were evaluated by a board-certified anesthesiologist as part of each patient’s preoperative assessment.^10^ Patients were divided into 2 groups, with the included cases being separated into either the low ASA class group (ASA score ≤2) or the high ASA class group (ASA score ≥ 3) for further analysis based upon their assigned ASA classification score.

PROMs, including the ODI^15^ and EQ-5D,^16^ were investigated prior to vertebroplasty, as well as after surgery at the 1-month, 3-month, 6-month and 12-month follow-up dates. The Japanese EQ-5D-3 L value set^5^ was used to assess quality of life. Patients who had missing information with regards to their PROMs were excluded from the study. The questionnaire for baseline PROMs was conducted by a trained nurse on the day before surgery. A follow-up assessment was conducted either at the outpatient department or via phone calls during the 1-month (3‒5 weeks), 3-month (2‒4 months), 6-month (5‒7 months) and 12-month (11‒13 months) follow-up dates. ODI scores are used to indicate the disability that is directly related to one’s back pain. A higher ODI score implies a greater severity of diability. The EQ-5D is a standardized measurement of health-related quality of life, which consists of five dimensions, including mobility, self-care, usual activities, pain and discomfort, and anxiety and depression. A higher total EQ-5D value indicates a better health status. Long term severe outcomes of patients’ survival status were investigated in the study as well. All-cause mortality of the included patients was confirmed through March 2022 using data obtained from the Ministry of Health and Welfare, R.O.C. Thereafter, de-identified data were used for analyses.

### Statiscal analysis

All of the statistical analyses were performed using the Statistical Package for the Social Sciences (IBM SPSS version 22.0; International Business Machines Corp, NY, USA). Categorical and continuous variables are presented as number (percentage) and mean ± SD, respectively. Between-group differences in clinical variables were tested for statistical significance using the independent Student’s *t*-test for continuous variables, and the χ^2^ test for categorical variables. Differences between the baseline and follow-up values of ODI and EQ-5D were compared using the paired Student’s *t*-test. Multivariable linear regression analyses were performed to evaluate the associations between preoperative ASA score (≥ 3 vs. ≤ 2) and changes in ODI and EQ-5D from baseline to 12-months. Kaplan-Meier survival curves were plotted for the study participants according to their preoperative ASA classification (≥ 3 vs. ≤ 2). The association of preoperative ASA classification with all-cause mortality was examined using Cox-proportional hazard models with adjustment for age, gender, baseline ODI and EQ-5D, and osteoporosis. A two-sided p-value of <0.05 was considered statistically significant.

## Results

**Fig. 1:**
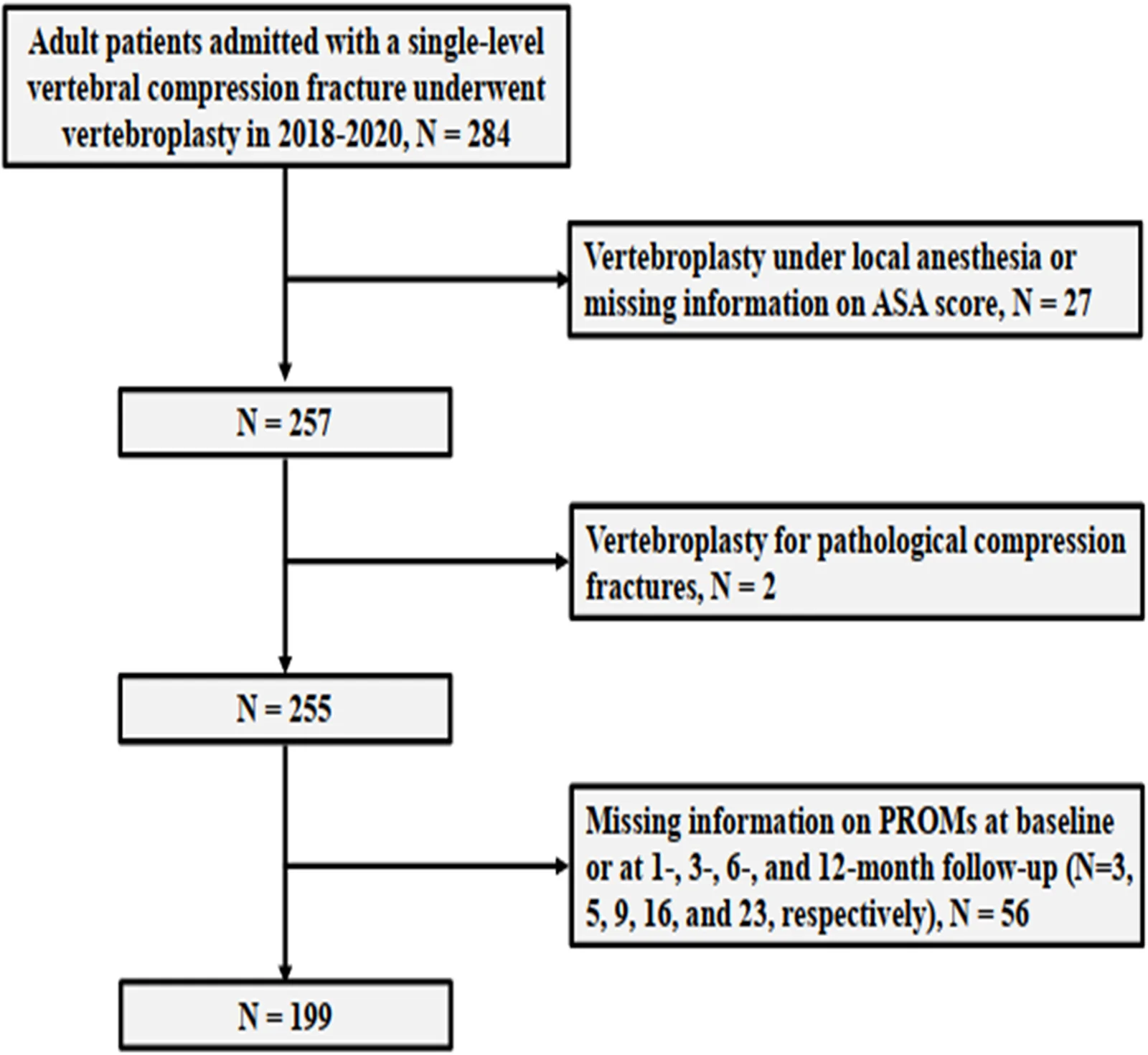
Flow diagram showing the number of patients identified, excluded, and those included in the final analysis. A total of 199 patients with complete PROMs data at baseline and at all follow-up visits were included in the analysis. ASA, the American Society of Anesthesiologists; PROM, Patient-Reported Outcome Measure.

Fig. 1 shows the flow diagram displaying the number of patients identified, excluded, and those included in the final analysis. A total of 199 patients with complete PROMs data at baseline and at all follow-up visits were included in the analysis, of which 95 patients had a preoperative ASA ≤ 2 and 104 had an ASA ≥ 3 (Table 1). Patients with an ASA ≥ 3 were shown to be older (mean [SD] age 78.6 [9.2] vs. 72.4 [9.0] years, p < 0.001), had a higher proportion of hypertension (64.4%vs. 40.0%, p = 0.001) and chronic kidney disease (28.8%vs. 12.6%, p = 0.005), as well as a higher preoperative ODI (mean [SD] 73.5 [11.9] vs. 68.2 [15.0], p = 0.007) than those who had an ASA ≤ 2. There were no significant between-group differences in the other variables.

**Table 1:** Baseline characteristics of the study patients through preoperative ASA classification.

	**Preoperative ASA classification**		
	**≤ 2**	**≥ 3**	**p**
**Number of patients**	95	104	
**Age, years**	72.4 (9.0)	78.6 (9.2)	<0.001
**Male gender, n (%)**	19 (20.0)	30 (28.8)	0.148
**Body mass index, kg/m^2^**	24.0 (3.7)	24.5 (4.3)	0.468
**Smoking, n (%)**	3 (3.2)	8 (7.7)	0.162
**Diabetes, n (%)**	18 (18.9)	28 (26.9)	0.183
**Hypertension, n (%)**	38 (40.0)	67 (64.4)	0.001
**Chronic kidney disease, n (%)**	12 (12.6)	30 (28.8)	0.005
**Osteoporosis, n (%)**	70 (73.7)	73 (70.2)	0.584
**Level of vertebroplasty, n (%)**			0.904
T-spine	41 (43.2)	44 (42.3)	
L-spine	54 (56.8)	60 (57.7)	
**Oswestry Disability Index**	68.2 (15.0)	73.5 (11.9)	0.007
**EuroQol-5D**	0.26 (0.22)	0.23 (0.23)	0.424

Values are mean (SD) or n (%).

ASA, the American Society of Anesthesiologists.

**Fig. 2:**
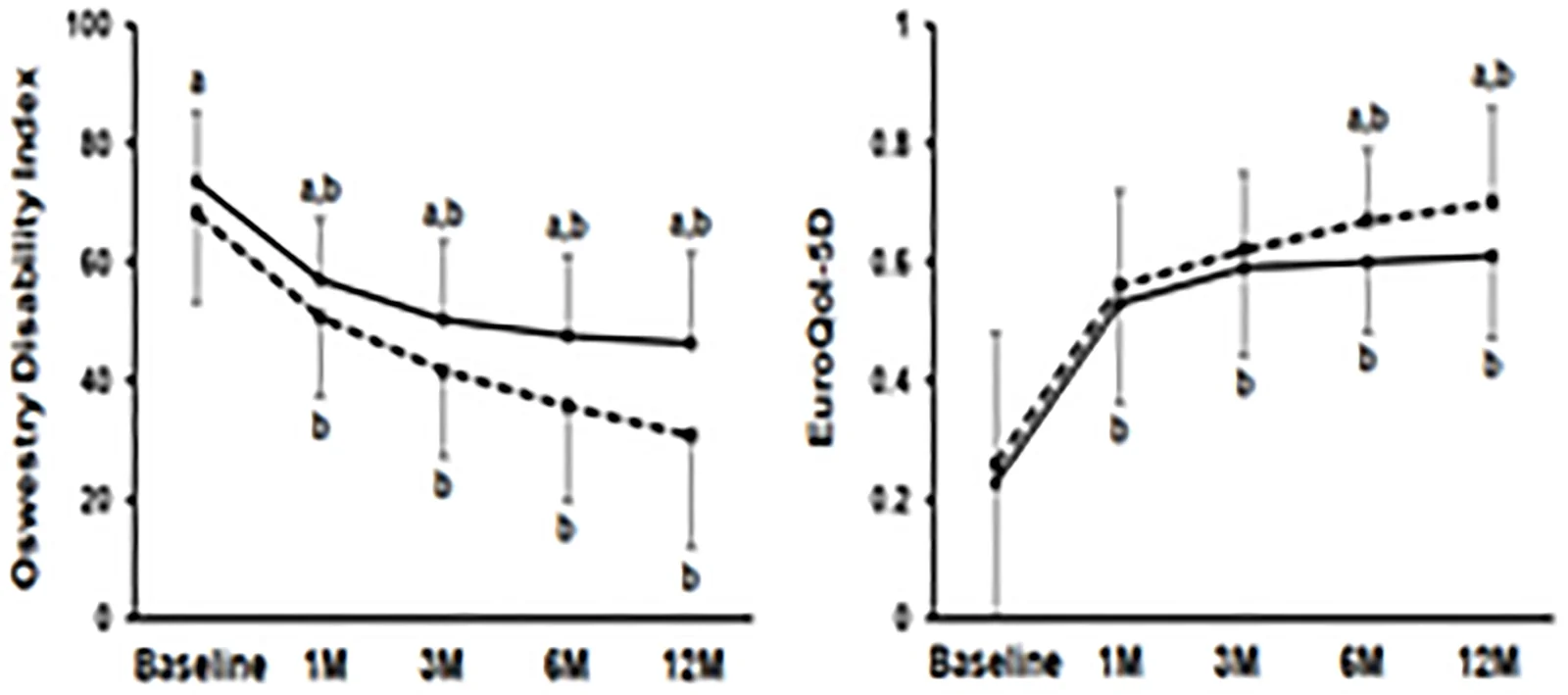
Oswestry Disability Index (left panel) and EuroQol-5D (right panel) at baseline and at each follow-up time point according to preoperative ASA classification. Data are presented as the mean ± standard deviation (error bars). Solid line, ASA ≥ 3. Dashed line, ASA ≤ 2. ASA, the American Society of Anesthesiologists. ^a^ p < 0.01 for between-group differences. ^b^ p < 0.001 vs. baseline.

Fig. 2 shows ODI and EQ-5D at baseline and at each follow-up time point after vertebroplasty treatment according to preoperative ASA classification. ODI significantly decreased and EQ-5D significantly improved after surgery in both groups. At the 12-month follow-up period, patients with an ASA ≥ 3 had a higher ODI and a lower EQ-5D than those with an ASA ≤ 2. Table 2 shows changes from baseline in ODI and EQ-5D during the follow-up period. Patients with an ASA ≤ 2 experienced a larger reduction in ODI (mean [SD] −35.8 [18.7] vs. −25.5 [17.4], p = 0.001) and a greater improvement in EQ-5D (mean [SD] 0.42 [0.22] vs. 0.33 [0.26], p = 0.042) at 12-month follow-up than those who had an ASA ≥ 3. Table 3 shows the associations of ASA classification (≥ 3 vs. ≤ 2) with changes in ODI and EQ-5D from baseline to 12-months of follow-up. We observed that a higher ASA score (≥ 3 vs. ≤ 2) was significantly associated with increased ODI (β-coefficient = 7.823, 95% CI 2.917 to 12.729, p = 0.002) and decreased EQ-5D (β-coefficient = −0.055, 95% CI −0.106 to −0.005, p = 0.031) from baseline to 12-months after multivariable adjustment.

**Table 2:** Changes from baseline in ODI and EQ-5D through preoperative ASA classification.

	**Preoperative ASA classification**		
	**≤ 2**	**≥ 3**	**p**
**ODI change from baseline**			
At 1-month follow-up	−17.3 (13.6)	−15.9 (12.2)	0.473
At 3-month follow-up	−25.5 (15.2)	−22.4 (14.6)	0.177
At 6-month follow-up	−31.0 (16.2)	−24.9 (15.1)	0.013
At 12-month follow-up	−35.8 (18.7)	−25.5 (17.4)	0.001
**EQ-5D change from baseline**			
At 1-month follow-up	0.30 (0.22)	0.28 (0.26)	0.669
At 3-month follow-up	0.35 (0.23)	0.34 (0.27)	0.755
At 6-month follow-up	0.40 (0.22)	0.36 (0.25)	0.237
At 12-month follow-up	0.42 (0.22)	0.33 (0.26)	0.042

Values are mean (SD). ASA, the American Society of Anesthesiologists; EQ-5D, EuroQol-5D; ODI, Oswestry Disability Index.

**Table 3:** Associations of preoperative ASA classification with changes in ODI and EQ-5D.

**Independent variable: ASA classification (≥ 3vs. ≤ 2)**	**β coefficient (95% CI)**	**p**
Dependent variable: ODI changes from baseline to 12-months		
Unadjusted model	10.388 (4.564, 16.212)	0.001
Adjusted model^a^	7.823 (2.917, 12.729)	0.002
Dependent variable: EQ-5D changes from baseline to 12-months		
Unadjusted model	−0.081 (−0.159, −0.003)	0.042
Adjusted model^b^	−0.055 (−0.106, −0.005)	0.031

ASA, the American Society of Anesthesiologists; EQ-5D, EuroQol-5D; ODI, Oswestry Disability Index.

a Adjusted for age, sex, baseline ODI, diabetes, hypertension, chronic kidney disease, and osteoporosis.

b Adjusted for age, sex, baseline EQ-5D, diabetes, hypertension, chronic kidney disease, and osteoporosis.

**Fig. 3:**
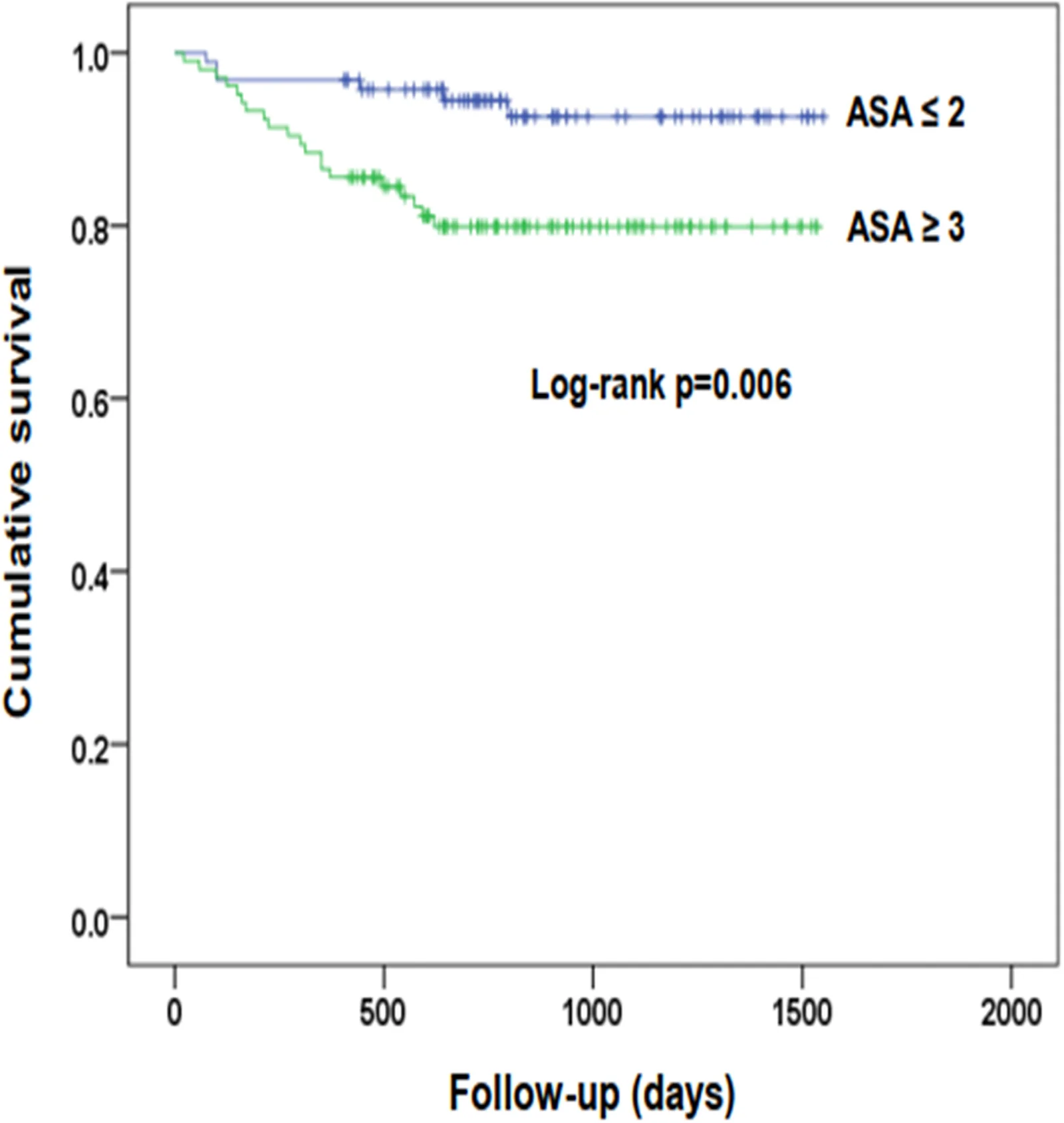
Kaplan-Meier survival curves according to preoperative ASA classification (ASA ≥ 3 vs. ASA ≤ 2). ASA, the American Society of Anesthesiologists.

Fig. 3 shows the Kaplan-Meier survival curves according to preoperative ASA classification (ASA ≥ 3 vs. ASA ≤ 2). Patients with an ASA ≥ 3 had a lower survival rate than those who had an ASA ≤ 2 (log-rank p = 0.006) during a median follow-up duration of 2.2-years. The association of preoperative ASA classification with all-cause mortality is examined in Table 4. Patients with an ASA ≥ 3 showed a higher risk of all-cause mortality compared with those who had an ASA ≤ 2 (HR = 3.315, 95% CI 1.330 to 8.258, p = 0.010). The association remained significant after multivariate adjustment (HR = 3.058, 95% CI 1.190 to 7.859, p = 0.020).

**Table 4:** Association of preoperative ASA classification with all-cause mortality.

**Independent variable**	**Hazard Ratio (95% CI)**	**p**
Preoperative ASA classification (≥ 3 vs. ≤ 2)		
Model 1	3.315 (1.330, 8.258)	0.010
Model 2	3.025 (1.173, 7.803)	0.022
Model 3	3.058 (1.190, 7.859)	0.020

ASA, the American Society of Anesthesiologists; Model 1, Unadjusted; Model 2, Adjusted for age and gender; Model 3, Adjusted for variables in Model 2 plus baseline Oswestry Disability Index, EuroQol-5D and osteoporosis.

## Discussion

There is a scarcity of research to date which specifically investigates the relationship between ASA classification and minimally invasive spinal procedures. In this single-center retrospective study, we found that there was significant improvement in self-reported outcomes, including both ODI and EQ-5D, after vertebroplasty for compression fractures in both low (ASA ≤ 2) and high (ASA ≥ 3) ASA groups. Nevertheless, greater improvement in the self-reported outcome measures at 12-month follow-up was noted in the former group (Fig. 2 and Table 2). We also observed a significantly higher risk of mortality in patients with a higher preoperative ASA (≥ 3 vs. ≤ 2) (Fig. 3 and Table 4). Our findings suggest that preoperative ASA was associated with postoperative changes in self-reported outcome measures and long-term mortality in patients who had undergone vertebroplasty for compression fracture.

Along with the growth of aging populations throughout the world, the prevalence of osteoporosis has significantly increased in recent decades.^17^ Among patients with osteoporosis, vertebral compression fracture is a common complication which may lead to pain and disability, a decrease in life quality, an increase in healthcare cost and even mortality.^18^^,^^19^ Vertebroplasty has been considered as a means to both relieve pain and improve physical function in patients with vertebral compression fracture.^3^^,^^20^ However, the effects of vertebroplasty on the aforementioned outcomes,^21^ as well as certain patient-reported outcome measures,^22^, ^23^, ^24^ have not yet been made clear. PROMs have become increasingly important. Traditional outcome measures such as complications and survival rates for measurement may not provide a complete picture of patients’ postoperative experiences.^25^ ODI and EQ-5D are two of the most commonly used PROMs used after orthopedic surgeries.^6^^,^^26^^,^^27^ Both of them have shown solid reliability in estimating one’s health state values, particularly after lower back surgery.^28^ We found that ODI and EQ-5D significantly improved after vertebroplasty (Fig. 2), and that preoperative ASA was associated with postoperative changes in these outcome measures (Table 2). Similar findings were noted in patients who had undergone surgery for hip fracture^29^ (acute trauma) as well as those who had undergone total hip replacement (elective surgery).^30^ This observation suggests that baseline physiological reserve, as reflected by the ASA score, is a key determinant of postoperative recovery, regardless of whether the procedure is an emergency trauma surgery or an elective orthopedic operation. Although these surgeries may vary in clinical scenarios, the aforementioned results suggest that preoperative ASA may help identify patients who may experience a greater improvement in patient-reported outcomes after orthopedic surgery.

Nevertheless, findings on the effects of surgical interventions for vertebral fractures on mortality are inconsistent.^31^, ^32^, ^33^, ^34^ Additional studies are still required in order to better elucidate the benefits of vertebroplasty for compression fracture on long-term survival.^35^^,^^36^ ASA classification is a commonly used preoperative assessment tool to evaluate anesthesia risk. However, there is only limited available research on the correlation between ASA classification and postoperative outcomes, particularly in minor procedures. According to an analysis performed by Colin Foley et al., ASA was found to be a reliable predictor of both post-operative complications and mortality in outpatient surgeries.^37^ Regarding spinal surgery, several studies have explored the relationship between ASA classification and post-operative outcomes, including issues such as complication rates,^38^ blood transfusion risk,^39^ morbidity and mortality.^13^^,^^40^ Our findings suggest that preoperative ASA classification is associated with postoperative long-term outcomes, even in less invasive surgeries.

The strengths of this study include the use of both a disease-specific (ODI) and a generic (EQ-5D) PROM, the inclusion of hard endpoint (mortality) data, and rigorously conducted statistical analyses, which together provide a comprehensive picture of patient outcomes. However, several limitations of this study should be acknowledged. First, it was a retrospective study. Second, the relatively small sample size and use of only a single-center may have limited the generalizability of our findings. Third, patients’ responses to baseline PROM assessments (conducted on the day before surgery) may be influenced by the anticipation of a stressful procedure. Fourth, we excluded patients with missing PROM data at baseline or at follow-up from the analyses (n = 56). Potential survivor bias should be considered when interpreting our results. The baseline characteristics of these patients (n = 56) were as follows: mean age 74.6 ± 8.9 years, male 25%, mean body mass index 24.3 ± 5.1 kg/m^2^, smoking 8.9%, diabetes 17.9%, hypertension 64.3%, and chronic kidney disease 21.4%. Compared with those included in the analysis (n = 199), there were no significant differences in the aforementioned baseline characteristics (all p > 0.05). Last but not the least, the majority of our patients were of ASA class 2 or class 3 (97.0%). ASA class 1 represents healthy individuals, while ASA class 4 or higher denotes patients with incapacitating diseases or even life-threatening conditions. These patients represent a minor proportion of those who eventually undergo spinal surgery. Future multi-center studies involving a larger sample size are still necessary in order to confirm our findings.

## Conclusions

We found that a lower ASA classification (≤ 2) was associated with a better improvement in PROMs after vertebroplasty treatment for compression fracture, when compared to those having an ASA ≥ 3. The former also experienced a better long-term survival rate. Our findings suggest that ASA classification may help surgeons identify patients at high risk for poor postoperative recovery, thereby facilitating more effective preoperative counseling regarding expected recovery trajectories and enabling the implementation of more intensive perioperative management strategies, even for patients undergoing less invasive procedures such as vertebroplasty.

## Abbreviations

ASA, American Society of Anesthesiologists; EQ-5D, EuroQol-5D; ODI, Oswestry Disbility Index; PROM, Patient-Reported Outcome Measure.

## Declaration of generative AI and AI-assisted technologies

Nothing to disclose.

## Authors’ contributions

Chin-Chiu Chen: Conceptualization; Methodology; Investigation; Writing-original draft. Heng-Hao Liu, Kun-Hui Chen, Ching-Heng Lin, and Cheng-Hung Lee: Data curation; Methodology; Investigation; Writing-review & editing. Jun-Sing Wang: Conceptualization; Data curation; Formal analysis; Methodology; Investigation; Validation; Resources; Project administration; Supervision; Writing-original draft.

## Funding

This study was supported by 10.13039/501100010101Taichung Veterans General Hospital, Taichung, Taiwan [grant numbers TCVGH-1123502C, 2023; TCVGH-1133503C, 2024]. The funder was not involved in the study design, data collection, analysis, interpretation of the results, or preparation of the article.

## Declaration of competing interest

The authors declare no conflicts of interest.

## Data Availability

The datasets used and/or analyzed during the current study are available from the corresponding author on reasonable request.

## References

[1] Lin Y.Y, Huang C.S. (2016). Aging in Taiwan: building a Society for Active Aging and Aging in place. Gerontologist.

[2] Lin J.T, Lane J.M. (2004). Osteoporosis: a review. Clin Orthop Relat Res.

[3] Alsoof D., Anderson G., McDonald C.L, Basques B., Kuris E., Daniels A.H. (2022). Diagnosis and management of vertebral compression fracture. Am J Med.

[4] Fairbank J.C, Pynsent P.B. (2000). The Oswestry disability index. Spine.

[5] Tsuchiya A., Ikeda S., Ikegami N., Nishimura S., Sakai I., Fukuda T. (2002). Estimating an EQ-5D population value set: the case of Japan. Health Econ.

[6] McCormick J.D, Werner B.C, Shimer A.L. (2013). Patient-reported outcome measures in spine surgery. J Am Acad Orthop Surg.

[7] Evans A.J, Kip K.E, Boutin S.M (2009). Prospective assessment of pain and functional status after vertebroplasty for treatment of vertebral compression fractures. J Neurointerv Surg.

[8] Santiago F.R, Abela A.P, Alvarez L.G, Osuna R.M, García Mdel M. (2010). Pain and functional outcome after vertebroplasty and kyphoplasty. A comparative study. Eur J Radiol.

[9] Xie L., Zhao Z.G, Zhang S.J, Hu Y.B. (2017). Percutaneous vertebroplasty versus conservative treatment for osteoporotic vertebral compression fractures: an updated meta-analysis of prospective randomized controlled trials. Int J Surg.

[10] American Society of Anesthesiologists. Statement on ASA Physical Status Classification System, https://journals.lww.com/anesthesiologyopen/fulltext/2026/01000/american_society_of_anesthesiologists_statement_on.2.aspx [accessed 20 December 2025].

[11] Daabiss M. (2011). American Society of Anaesthesiologists physical status classification. Indian J Anaesth.

[12] Whitmore R.G, Stephen J.H, Vernick C., Campbell P.G, Yadla S., Ghobrial G.M (2014). ASA grade and charlson comorbidity index of spinal surgery patients: correlation with complications and societal costs. Spine J.

[13] Fu K.M, Smith J.S, Polly D.W, Ames C.P, Berven S.H, Perra J.H (2011). Correlation of higher preoperative American Society of Anesthesiology grade and increased morbidity and mortality rates in patients undergoing spine surgery. J Neurosurg Spine.

[14] Somani S., Di Capua J., Kim J.S, Phan K., Lee N.J, Kothari P. (2017). ASA classification as a risk stratification tool in adult spinal deformity surgery: a study of 5805 patients. Glob Spine J.

[15] Fairbank J.C, Couper J., Davies J.B, O'Brien J.P (1980). The Oswestry low back pain disability questionnaire. Physiotherapy.

[16] Rabin R., de Charro F. (2001). EQ-5D: a measure of health status from the EuroQol group. Ann Med.

[17] Reginster J.Y, Burlet N. (2006). Osteoporosis: a still increasing prevalence. Bone.

[18] Compston J.E, McClung M.R, Leslie W.D. (2019). Osteoporosis. Lancet.

[19] Lavelle W., Carl A., Lavelle E.D, Khaleel M.A. (2007). Vertebroplasty and kyphoplasty. Med Clin North Am.

[20] Alvarez L., Alcaraz M., Pérez-Higueras A., Granizo J.J, de Miguel I., Rossi R.E (2006). Percutaneous vertebroplasty: functional improvement in patients with osteoporotic compression fractures. Spine (Phila Pa 1976).

[21] Buchbinder R., Johnston R.V, Rischin K.J, Homik J., Jones C.A, Golmohammadi K. (2018). Percutaneous vertebroplasty for osteoporotic vertebral compression fracture. Cochrane Database Syst Rev.

[22] Diel P., Reuss W., Aghayev E., Moulin P., Röder C. (2010). SWISSspine: a nationwide health technology assessment registry for balloon kyphoplasty: methodology and first results. Spine J.

[23] Lee H.M, Park S.Y, Lee S.H, Suh S.W, Hong J.Y. (2012). Comparative analysis of clinical outcomes in patients with osteoporotic vertebral compression fractures (OVCFs): conservative treatment versus balloon kyphoplasty. Spine J.

[24] Hübschle L., Borgström F., Olafsson G., Röder C., Moulin P., Popp A.W (2014). Real-life results of balloon kyphoplasty for vertebral compression fractures from the SWISSspine registry. Spine J.

[25] Birkmeyer J.D, Dimick J.B, Birkmeyer N.J. (2004). Measuring the quality of surgical care: structure, process, or outcomes?. J Am Coll Surg.

[26] Yu H.M, Malhotra K., Butler J.S, Patel A., Sewell M.D, Li Y.Z (2016). The relationship between spinopelvic measurements and patient-reported outcome scores in patients with multiple myeloma of the spine. Bone Jt J.

[27] Finkelstein J.A, Schwartz C.E. (2019). Patient-reported outcomes in spine surgery: past, current, and future directions. J Neurosurg Spine.

[28] Solberg T.K, Olsen J.A, Ingebrigtsen T., Hofoss D., Nygaard O.P. (2005). Health-related quality of life assessment by the EuroQol-5D can provide cost-utility data in the field of low-back surgery. Eur Spine J.

[29] Teni F.S, Burström K., Berg J., Leidl R., Rolfson O. (2020). Predictive ability of the American society of anaesthesiologists physical status classification system on health-related quality of life of patients after total hip replacement: comparisons across eight EQ-5D-3L value sets. BMC Musculoskelet Disord.

[30] Chen L.H, Liang J., Chen M.C, Wu C.C, Cheng H.S, Wang H.H (2017). The relationship between preoperative American society of anesthesiologists physical status classification scores and functional recovery following hip-fracture surgery. BMC Musculoskelet Disord.

[31] Edidin A.A, Ong K.L, Lau E., Kurtz S.M. (2013). Life expectancy following diagnosis of a vertebral compression fracture. Osteoporos Int.

[32] McCullough B.J, Comstock B.A, Deyo R.A, Kreuter W., Jarvik J.G. (2013). Major medical outcomes with spinal augmentation vs conservative therapy. JAMA Intern Med.

[33] Edidin A.A, Ong K.L, Lau E., Kurtz S.M. (2011). Mortality risk for operated and nonoperated vertebral fracture patients in the medicare population. J Bone Min Res.

[34] McDonald, R.J, Achenbach, S.J, Atkinson, E.J, Gray, L.A, Cloft, H.J, LJM, I.II., et al. Mortality in the vertebroplasty population. 2011;32(10):1818–23. 10.3174/ajnr.A2616 PMC3217093 21998109

[35] Diamond T.H, Bryant C., Browne L., Clark W.A (2006). Clinical outcomes after acute osteoporotic vertebral fractures: a 2-year non-randomised trial comparing percutaneous vertebroplasty with conservative therapy. Med J Aust.

[36] Láinez Ramos-Bossini A.J, López Zúñiga D., Ruiz Santiago F. (2021). Percutaneous vertebroplasty versus conservative treatment and placebo in osteoporotic vertebral fractures: meta-analysis and critical review of the literature. Eur Radiol.

[37] Foley C., Kendall M.C, Apruzzese P., De Oliveira G.S. (2021). American society of anesthesiologists physical status classification as a reliable predictor of postoperative medical complications and mortality following ambulatory surgery: an analysis of 2089,830 ACS-NSQIP outpatient cases. BMC Surg.

[38] Schoenfeld A.J, Carey P.A, Cleveland A.W, Bader J.O, Bono C.M. (2013). Patient factors, comorbidities, and surgical characteristics that increase mortality and complication risk after spinal arthrodesis: a prognostic study based on 5887 patients. Spine J.

[39] Shillingford J.N, Laratta J.L, Lombardi J.M, Mueller J.D, Cerpa M., Reddy H.P (2018). Complications following single-level interbody fusion procedures: an ACS-NSQIP study. J Spine Surg.

[40] Pateder D.B, Gonzales R.A, Kebaish K.M, Cohen D.B, Chang J.Y, Kostuik J.P. (2008). Short-term mortality and its association with independent risk factors in adult spinal deformity surgery. Spine.

